# The regulation of the pedunculopontine tegmental nucleus in sleep–wake states

**DOI:** 10.1007/s41105-023-00489-7

**Published:** 2023-09-26

**Authors:** Yiting Luo, Ying Li, Jie Yuan

**Affiliations:** 1 Department of Anesthesiology, The Affiliated Hospital of Zunyi Medical University, No.149 Dalian Road, Huichuan District, Zunyi, 563000 Guizhou China; 2 Department of Pain Medicine, Affiliated Hospital of Zunyi Medical University, Zunyi, 563000 Guizhou China; 3 Guizhou Key Laboratory of Anesthesia and Organ Protection, Zunyi Medical University, Zunyin, China

**Keywords:** PPTg, Sleep–wake, Cholinergic neurons, Glutamatergic neurons, GABAergic neurons

## Abstract

The pedunculopontine tegmental nucleus (PPTg) plays a vital role in sleep/wake states. There are three main kinds of heterogeneous neurons involved: cholinergic, glutamatergic, and gamma-aminobutyric acidergic (GABAergic) neurons. However, the precise roles of cholinergic, glutamatergic and GABAergic PPTg cell groups in regulating sleep–wake are unknown. Recent work suggests that the cholinergic, glutamatergic, and GABAergic neurons of the PPTg may activate the main arousal-promoting nucleus, thus exerting their wakefulness effects. We review the related projection pathways and functions of various neurons of the PPTg, especially the mechanisms of the PPTg in sleep–wake, thus providing new perspectives for research of sleep–wake mechanisms.

## Introduction

Arousal is an essential state for human survival, thus ensuring brain function. Sleep is the advanced physiological activity of life, which is affected by the steady state of the internal environment and the diurnal rhythm [1]. The states of sleep and wakefulness and the transition between them require very complex brain networks to regulate [2, 3]. With the progress and development of society, the common use of electronic products and the high intensity of work. Sleep disorders have always been of great concern to society. Sleep disorders are the manifestation of an abnormal amount of sleep and abnormal behavior during sleep, and also the manifestation of a disturbance of the normal rhythm of sleep and wakefulness. It is also a manifestation of disruption of the normal rhythm of sleep and wakefulness. When the brain switches from wake to sleep, the conscious activity weakens and even fades away. There is a transition from fast oscillatory low-amplitude activity to low oscillatory high-amplitude activity from wake to sleep, characteristic of cerebral cortical activation, electroencephalogram (EEG) “desynchronization” and arousal [4, 5]. A recent study finds that the brain nervous system can regulate the generation and duration of sleep, rapid eye movement (REM) sleep, and non-REM sleep in an orderly way [3, 4]. Sleep–wake regulation is controlled by complex interactions between several groups of neurons, which show changes in solid firing rate dependent on the state of arousal [2]. Therefore, knowing the mechanisms of the neural circuit is vital to sleep–wake.

The pedunculopontine tegmental nucleus (PPTg) is a significant part of the ascending reticular activating system (ARAS) and has a wide range of neural projections in the brain, and is considered the critical point in the regulation of arousal and REM sleep. This is mainly due to the cholinergic neurons contained in the PPTg nucleus. However, the PPTg nucleus also contains glutamatergic and GABAergic neurons that likely contribute to the regulation of sleep–wake. However, the mechanisms are incompletely understood [6]. Maintenance and transition of sleep–wake is regulated by the central nervous system. Decoding the neural network of sleep–wake regulation is particularly important for treating sleep disorders and improving sleep quality. Therefore, the literature reviews mainly the physiological functions of the PPTg in sleep/wake states.

## Anatomical and physiological functions of the PPTg

The PPTg, located in the brainstem, has a complex and unique pattern of input–output relationships, allowing it to participate in multiple functions [7]. Intracellular and extracellular recordings show electrophysiologically different cell types in the PPTg [8]. One type is characterized burst firing neurons with low-threshold Ca spikes, the second type is fast firing neurons with short-duration spikes, and the third type is slow firing neurons with long-duration spikes. Previous studies have shown that the PPTg is rich in cholinergic neurons [9], but subsequent studies have also shown that glutamatergic neurons and GABAergic neurons also occupy a considerable part [10]. Anatomically, GABAergic neurons predominate in the rostral portion, whereas cholinergic and glutamatergic neurons are most abundant in their caudal part [11, 12]. Meanwhile, the PPTg is divided into the pars compacta and the pars dissipate. In the pars dissipate, the proportion of GABAergic neurons is the highest, about 40%, and the glutamatergic neurons in pars compacta constitute the majority, accounting for roughly half of them [10] (Table 1).

**Table 1:** Overview: anatomical and physiological functions of the PPTg

Neuron type	Proportion %	Inputs signals	Target regions	Behaviour
Cholinergic	31 ± 3^①^	LH; TMN; DRN LC; PPTg	Cerebral cortex; Cerebral nuclei; Thalamus; Hypothalamus; Midbrain; (PnO),	Sleep–wake Motor function Reward and learning
	23 ± 3^②^			
Glutamatergic	50 ± 4^①^		STN; SNc; SNr; BF; LH; VTA; ZI	Wakefulness reward Motor function
	37 ± 2^②^			
GABAergic	19 ± 2^①^		Hardly	Production and maintenance of sleep
	40 ± 4^②^			

*LH* lateral hypothalamus, *TMN* tuberomammillary nucleus, *DRN* dorsal raphe nucleus, *LC* locus coeruleus, *PPTg* pedunculopontine tegmental nucleus, *VTA* ventral tegmental area, *BF* basal forebrain, *SNc* substantia nigra pars compacta, *SNr* substantia nigra pars reticulata, *PnO* nucleus pontis oralis, *ZI* zona incerta, *STN* subthalamic nucleus

^①^The pars compacta

^②^The pars dissipate

The PPTg has a complex neural circuit in the brain. There are many regions of the brain that promote arousal innervated by the PPTg, including the ventral tegmental area (VTA), the lateral hypothalamus (LH) and the basal forebrain (BF), the frontal cortex, and many thalamic nuclei [13–18]. It receives inputs from the hypocretin/orexin (ORX) neurons of the posterior LH and histaminergic (HA) neurons of the tuberomammillary nucleus (TMN), serotonergic inputs from the dorsal raphe nucleus (DRN), noradrenergic input from the locus coeruleus (LC), cholinergic input from the contralateral PPTg [8, 19–26]. Furthermore, the PPTg receives projections from the motor cortex, the basal ganglia, substantia nigra pars reticulate (SNr) and globus pallidus internus (GPi), the subthalamic nucleus (STN), and the deep cerebellar nuclei [27, 28]. The PPTg also activates and innervates thalamocortical neuron activity through the dorsal pathway directly in thalamus by acting on nicotinic and muscarinic receptors, and indirectly in the thalamocortical neurons by acting on M2 receptors to promote the release of excitatory neurotransmitters, thus activating the cortex and maintaining cortical excitability [14, 29, 30]. Most of these inputs and outputs are significant in the regulation of sleep–wake. Meanwhile, PPTg has a tight fiber connection of numerous nuclei in the brain, which determines various functions, such as sleep–wake, motor function, consciousness, action selection, reward mechanisms, etc. [11, 31]. Recently, scholars have paid more attention to the role of the PPTg in sleep–wake [6, 13].

PPTg is the most active nuclei during waking and REM sleep [32]. But how the PPTg promotes wake remains unclear. Dautan et al.’s opinion is that the connections to dopaminergic nuclei receive excitatory input from the PPTg, thus declaring the PPTg plays a crucial role in arousal [33]. Steriade et al. [34] thought the cholinergic of the PPTg projections on thalamic activity. During wakefulness, acetylcholine facilitates thalamocortical signaling by directly exciting thalamocortical relay neurons while reducing activity in the reticular nucleus of the thalamus, which inhibits thalamocortical neurons. At the onset of non-REM sleep, reduced cholinergic activity has the opposite effects. Besides, high-frequency electrical stimulation of the PPTg in sleeping and anesthetized cats induces fast EEG patterns [35].The PPTg is also implicated in contributing the REM sleep. Previous studies show that many neurons in the PPTg are active during REM sleep [36–40]. Deurveilher et al. [41] found neurotoxic destruction of the PPTg changed the ability of animals to respond to challenges such as deprivation of REM sleep. In intermittently hypoxic rats, people found that the PPTg lesions changed the structural organization of rest, REM sleep decreased while non-REM sleep increased [42]. These studies suggest a role for the PPTg neurons in REM sleep control.

All of the above demonstrate the PPTg promoting wakefulness and controlling REM sleep. The PPTg can influence arousal through its effects on different subcortical regions and is also involved in maintaining REM sleep. It might go through the central neurons directly and indirectly, thus playing a role. Moreover, the three kinds of neurons in the PPTg may result in different effects.

## The role of various neurons in sleep–wake in the PPTg

### The cholinergic neurons of the PPTg in sleep–wake

Cholinergic neurons are the smallest group in the PPTg, accounting for 27% of the total number of neurons [10]. Acetylcholine (Ach) was proposed to be the main neurotransmitter of ARAS. The ascending middle cholinergic system (AMCS) is one of the main components of ARAS. AMCS is derived from the PPTg, which is derived from cholinergic cell bodies located primarily in the PPTg, mainly innervating the medial midbrain and basal forebrain regions. Systemic or intracerebral injection of cholinergic receptor agonists can generate pontine-geniculate-occipital (PGO) waves before the onset of REM sleep and throughout REM sleep [43]. PGO waves occur during and before REM sleep and are considered a neurophysiological hallmark of REM sleep in mammals [44]. Single cell tracking and reconstruction of cholinergic neurons reveal unique ways of their connections. The cell body is the origin of its axons. On average, there are five collaterals and most of them have ascending trajectories along dorsal and ventral streams [7, 45], and ascending ventral innervation is located in the basal ganglia and extends to the hypothalamic region of the BF. There are shreds of evidence showing that the synapses formed by cholinergic axons are located in the cerebral cortex, the cerebral nuclei, the thalamus, the hypothalamus, the midbrain, the pontine reticular formation (PnO), the medulla and the cerebellum [28]. Therefore, the level of collateralization in cholinergic neurons is high, which innervate the structures of the midbrain, forebrain, and lower brainstem. The PPTg, as one of the primary sources of cholinergic innervation of the thalamus and brainstem, is closely associated with the sleep–wake cycle.

The researcher has shown that the most medial portion of the PPTg cholinergic neuron is active during wakefulness and REM sleep [37]. Previous studies have also shown that activation of cholinergic neurons during non-REM sleep does not increase the duration of REM sleep, but the number of REM sleep episodes is increased [6]. All of the above indicate that the cholinergic neurons in the PPTg that are essential for the initiation of REM sleep, but are not crucial for the maintenance of REM sleep. Kroeger et al. discovered that cholinergic chemogenetics activation in the PPTg neurons does not affect REM sleep but promotes non-REM sleep a little [13]. By optogenetics and juxtacellular recording, Cissé et al. found that cholinergic neurons in PPTg promote θ and γ cortical activity during Wake and REM sleep [39].

Cholinergic neurons in the PPTg are involved in the modulation of several sleep–wake nuclei in the brain. HA neurons in TMN are important neurons arousal promoting arousal, TMN emits nerve fibers that project to the PPTg, which regulate the PPTg by activating receptors linked to the H1 and H2 G protein-coupled, and thus stimulate the cortex to facilitate awakening [46]. The cholinergic fibers in the PPTg projected into the arousal-promoting nucleus of the brainstem, such as PnO, and promote cortical activity [45]. Mena–Segovia et al. confirmed that the PPTg plays a role in the cortical transition from a slow-wave, low arousal state to a high arousal state [7]. The acetylcholine-releasing nucleus of the PPTg is mainly aimed at the BF, but also to the relay nucleus and reticular cells of the thalamus [28]. This pathway is essential to stimulate the transmission of thalamic signals to the cerebral cortex. In addition, ORX neurons in the posterior hypothalamus, tuberal hypothalamus, and LH areas innervate cholinergic neurons in PPTg via excitatory receptors (Orx1 and 2R) to produce arousal [47]. All the above shows that cholinergic neurons are closely related to the state of sleep and wakefulness in the cortical EEG. At the same time, cholinergic neurons in the PPTg also regulate the state of sleep through the input and output of multiple nuclei. Cholinergic neurons’ activity is higher during EEG desynchronization in cortical activity. Cholinergic neurons of the PPTg have been showed to fire at higher rates during waking and REM sleep than during non-REM sleep in the sleeping rat [37]. The firing of cholinergic neurons appears to be discontinuous and episodic [45]. In my opinion, it is this discontinuous and episodic that further confirms that the number of REM sleep episodes increased but not the duration of REM sleep.

The cholinergic neurons of the PPTg have an extensive range of neural projections in the brain, and their axons are highly collateralized. They play an essential role in sleep/wake states through tight connections with multiple regions of the brain. However, cholinergic neurons are not essentially required for the awake state. They are tightly linked to thalamocortical circuits, while thalamocortical activation is associated with cortical activity. Moreover, we think that the PPTg cholinergic neurons are necessary but not sufficient for sleep–wake. To some extent, the absence of the PPTg only influences little. The reason why this occurs is it that can be compensated by other nuclei, and the sleep–wake transition is the result of joint involvement of several nuclei in the brain.

### The glutamatergic neurons of the PPTg in sleep–wake

Glutamatergic neurons are the other vital neuron in PPTg. In the central nervous system, glutamate is the primary excitatory neurotransmitter. As a chemical signal, it plays an essential role in intercellular interactions and has a broad role in sleep–wake regulation. The PPTg glutamatergic neurons are active during wake and REM sleep [37]. In contrast, glutamate injection after NMDA antagonist pretreatment reduces wakefulness, and injection of KA receptor agonist only increases REM sleep [48]. It seems that glutamatergic neurons of the PPTg play a more critical role in electrophysiology than cholinergic neurons. Synapses formed by glutamatergic neurons originate in the basal ganglia [49]. Researchers demonstrated that glutamatergic neurons have higher firing rates during states of cortical activation and REM sleep than cholinergic neurons [37]. Kroeger et al. [13] found that selective activation of the PPTg glutamatergic neurons can induce longer behavioral arousal and cortical activation, while inhibition reduces arousal and increases non-REM sleep. This is consistent with a recent study by Kroeger et al., they found photoactivation of the PPTg glutamatergic neurons can wake mice from non-REM sleep, and photoactivation of distinct axonal projections can lead to differences in waking behavior [50]. Glutamatergic neurons in the PPTg neurons are composed of, in addition to neurons that are most active during waking (Wake active neurons), neurons that are active both during waking and REM sleep (Wake/REM active neurons) and those that are most active during REM sleep (REM active neurons) [37]. Microinjections of glutamate into the PPTg were found to increase waking and REM sleep [51] so glutamatergic neurons in the PPTg have the effect of enhancing wakefulness.

However, the specific pathway by which glutamatergic neurons promote arousal is not clear. Kroeger et al. found that the glutamatergic neurons of the PPTg projections to the SN may promote arousal, while projections to the BF and LH may promote higher arousal levels under dynamic conditions [50]. Some evidence showed that dopaminergic neurons (SNc) received glutamatergic neurons input directly from the PPTg neurons [52, 53]. Furthermore, activation of the PPTg can cause excitatory effects on SN neurons in rats. Retrograde tracing experiments indicate the presence of glutamatergic afferents from the PPTg to the VTA [53]. The glutamatergic link between the PPTg and the VTA originated from neurons expressing the vesicular glutamate transporter 2 (VGLUT2) [54]. It also found that the PPTg to the VTA connections were more abundant on the ipsilateral side, suggesting that noncholinergic links affect dopaminergic midbrain cell activity [55]. In rats, neurons sending projections to the VTA are concentrated in the caudal PPTg, project bilaterally, and involve glutamatergic axons [56]. These brain regions are believed to contribute to arousal, suggesting that glutamatergic neurons of the PPTg may promote arousal through complicated inputs and outputs.

This evidence suggests that glutamatergic neurons play a more critical role than cholinergic neurons in a cortically activated state. The part of the PPTg glutamatergic neurons in sleep/wake states are not apparent, and the specific input and output of the PPTg glutamatergic neurons are not well understood. It is undeniable that the activation of the PPTg glutamatergic neurons may be sufficient to keep the mice awake. Still the involvement of more wake-promoting brain regions may lead to higher levels of arousal.

### The GABAergic neurons of the PPTg in sleep–wake

The GABAergic type of the PPTg is another necessary neuron type. In the central nervous system, GABA is an inhibitory amino acid neurotransmitter involved in controlling neuronal activity. GABA passes through two types of receptor [57]. Ionotropic GABAA and GABAC and metabotropic GABAB receptors mediate their effects. In the nervous system, GABAB receptors are widely expressed, regulate synaptic excitability and plasticity in the cerebral cortex, generate rhythmic activity in the cortical and thalamic circuits, transmit primary input activity to the spinal cord and brainstem, and affect dopamine activity in neurons and other monoaminergic neurons [57]. A study found two types of GABAergic neurons in the PPTg, one of which discharges maximally during wakefulness and REM sleep, while the other discharges maximally during REM sleep [37]. Currently, the role of GABAergic neurons in the production and maintenance of sleep has been widely accepted. Kroeger et al. [13] found that chemogenetic activation of GABAergic in PPTg neurons reduced REM sleep. Cortical activation is characterized by rhythmic theta activity (6–10 Hz) in the limbic cortex [58]. Research found that optogenetic stimulation and recording of GABAergic neurons in the PPTg can discharge in rhythmic bursts at theta frequencies and drive theta activity in limbic cortex [59]. Similarly, to glutamatergic neurons, dopaminergic neurons (SNc) can also receive GABA input directly from the PPTg meridians in rats and monkeys [53]. In rats, neurons sending projections to the VTA are concentrated posterior to the PPTg, project bilaterally, and involve GABAergic axons [56]. However, recent studies have shown that the GABAergic neurons of the PPTg project more in situ [13].

In conclusion, the role of the PPTg GABAergic neurons in sleep/wake states appears limited. Activation of GABAergic neurons of the PPTg slightly reduces REM sleep. We know that in the PPTg, the proportion of GABAergic neurons is large, but its role in sleep–wake states is not as prominent as that of cholinergic and glutamatergic neurons. The most important reason is that its axonal collateralization is low and the projection is not extensive. Cortical activity and sleep/wake states are complex and sophisticated processes that require multiple regions of the brain work together.

## The other functions of the PPTg

The functions of the PPTg also include motor structures. Studies have found that the PPTg regulates muscle tension during wakefulness and muscle relaxation during NREM sleep. Studies have shown that the thalamic motor area includes the cuneiform nucleus and the PPTg. Both the PPTg and the cuneiform nucleus, glutamatergic neurons are significantly innervated from the basal ganglia, the amygdala, and the laterodorsal tegmental nucleus (LDT) [60]. Optogenetic stimulation of the PPTg glutamatergic neurons in VGluT2-cre mice induces locomotion [49]. Loss of the PPTg cholinergic neurons is also found in Parkinson’s disease, multiple system atrophy, and progressive supranuclear palsy. All of the above studies have shown that the PPTg affects motor function. The possible mechanism is that the pallidus globus is overactivated, releasing GABAergic transmitters and projecting to the glutamatergic neurons of the PPTg, reducing their activity and cause abnormal movement.

The PPTg plays a vital role in the regulation of dopamine neurons. A previous study found that the PPTg strongly modulates the SNc and the VTA neurons [61]. Other studies have demonstrated that the PPTg exit synaptic contacts with dopaminergic and non-dopaminergic neurons in the midbrain. The PPTg is the source of cholinergic innervation of dopaminergic neurons in the middle brain [33], and the dopaminergic midbrain and cholinergic brain stem are closely related to behavioral expression. This suggests that the PPTg is involved in the reward and plays a significant role.

The PPTg, the primary source of cholinergic projections in the brainstem, the cholinergic system plays a vital role in analgesia. In some brain structures (such as the hippocampus, DRN, and LC), microinjection of muscarinic and nicotinic cholinergic receptor antagonists can affect regulation of analgesia [62–64]. Its inputs play a relevant role in the nociceptive modulation of convulsive responses. For example, local injection of cobalt chloride into the PPTg in epileptic rats can deactivate synapses and cause no anticonvulsant effect. However, it can reduce the induced antinociceptive response, thus reducing the post-analgesic impact, suggesting that the PPTg is involved in the regulation of analgesia epilepsy induction [65]. In addition, studies have shown that with cholinergic agonists, the pharmacological manipulation of the PPTg can induce antinociception [66]. This suggests that the PPTg is involved in the analgesic and plays a significant role.

## Conclusions and prospects

The PPTg is essential for the regulation of sleep–wake. Various types of neurons in the PPTg and their related projection pathways contribute in a distinct way to the control of the sleep/wake states. The proportion of cholinergic neurons in the PPTg is small but significant. The above shows that cholinergic neurons have denser innervation than the other two types of neurons. We speculate that regulation of the PPTg function may depend primarily on cholinergic neurons. Multiple targets are reached and the activation of subsets of GABAergic or glutamatergic neuron can depend on various factors, including local and afferent modulation. As a result, its role is not as prominent as cholinergic neurons. Cholinergic neurons control thalamocortical excitability and behavioral states, whereas non-cholinergic neurons are more directly involved in regulating motor function. Activation of the PPTg glutamatergic neurons induces prolonged cortical activation and behavioral arousal, but inhibition reduces wakefulness and can increase non-REM sleep. Activation of the PPTg cholinergic neurons suppressed low-frequency EEG rhythms during non-REM sleep. Activation of the PPTg GABAergic neurons slightly reduces REM sleep. However, the projected relationship between the PPTg and other sleep–wake nuclei and the specific regulatory mechanism of sleep–wake has not been elucidated. We also do not fully understand whether the different types of neurons that regulate the sleep/wake states are independent or interact with one another. There is no doubt that the PPTg has a complex structure, but the effects of various regions on sleep and wakefulness are not completely clear; the neurons in the PPTg are heterogeneous, and the regulatory effects of these different neurons on sleep and wakefulness have not been thoroughly studied. The PPTg and other nerve nuclei and the network of relations between the nuclei and the circuit are huge, and there are still many unexplored aspects in current research. As for such a multifunctional nucleus, we know very little. Therefore, only by gradually clarifying these issues can we make significant progress in sleep–wake research and provide innovative ideas for the sleep–wake mechanism.
